# *Plasmodium falciparum* lacking histidine rich protein 2 and 3 genes in Colombia

**DOI:** 10.1186/1475-2875-11-S1-P27

**Published:** 2012-10-15

**Authors:** Erika Dorado, Claribel Murillo, Madeline Montenegro

**Affiliations:** 1 International Center for Medical Research and Training (CIDEIM), Cali, Colombia, 760031

## Background

Histidine rich protein 2 (HRP2) produced by *Plasmodium falciparum* is the target of most *P. falciparum-detecting* Rapid Diagnostic Tests (RDTs) as well as of *P. falciparum in vitro* drug susceptibility assays. Likewise histidine rich protein 3 (HRP3) is recognized to a minor proportion. Deletion of either *pfhrp2* or *pfhrp3* or both genes have been reported in *P. falciparum* parasites and can affect the RDT’s expected performance [1,2].

## Materials and methods

275 blood samples of P. *falciparum* collected on filter paper from 2003 to 2012 from seven malaria endemic localities in Colombia were evaluated. The presence of the *pfhrp2* and *pfhrp3* genes was tested by nested PCR amplification of two fragments of both genes. In addition flanking genes of *pfhrp2/pfhrp3* genes were evaluated. All the samples were confirmed as *P. falciparum* mono-infection.

## Results

15/275 (5.5%) samples showed a lack of the *pfhrp2* gene, all the samples come from the Amazon region. Preliminary results on *pfhrp3* gene showed that 98/275 (35.6%) of the samples were lacking this gene. 14/31 (45.2%) of the samples from the Amazon region were lacking both genes. Samples lacking *pfhrp2* and/or *pfhrp3* genes were missing either one or both flanking genes.

## Conclusions

The use of RDT’s based on the detection of HRP2 should be re-evaluated in the Amazon region due to the high level of parasites lacking the *pfhrp2* gene (48.4%). Monitoring the spread of *P. falciparum* lacking *pfhrp2/pfhrp3* genes should continue in South America.
